# Digital posters for interactive cellular media and bioengineering education

**DOI:** 10.1038/s42003-019-0702-1

**Published:** 2019-12-06

**Authors:** Mythreye Venkatesan, Ahmet F. Coskun

**Affiliations:** 1 Wallace H. Coulter Department of Biomedical Engineering, Georgia Institute of Technology and Emory University, Atlanta, GA USA; 2 Interdisciplinary Program in Bioengineering, Georgia Institute of Technology, Atlanta, GA USA; 3 School of Electrical and Computer Engineering, Georgia Institute of Technology, Atlanta, GA USA

**Keywords:** Education, Imaging, Software, Single-cell imaging, Molecular medicine

## Abstract

Conventional posters are effective in disseminating progress reports in scientific meetings, but they fail to deliver the need for visualization of dynamic biological data and become costly with the increasing number of conferences and the reprinting needs for emerging research. Here we present digital posters that repurpose digital frames from the art community and experiment with multiplexed imaging movies of cells as a demonstration of the digital poster concept, providing an interactive and low-cost tool for next-generation sharing platforms.

## Abstract (web summary)

Venkatesan and Coskun propose the use of digital posters instead of paper posters for disseminating progress reports in scientific meetings. These digital frames are capable of uploading multiple display items via wireless network and changing the interactive display with wave of a hand over an attached motion sensor.

## Revisiting poster designs for interactive media

Posters are indispensable in sharing research reports in scientific meetings and conferences. Classically, large format, high-quality, and expensive printed papers have been preferred for posters. Paper posters have covered introduction, methods, results, and conclusion sections under a research theme either for an individual, or a research team, or an academic center. Concise, visual, and visionary media have been highlighted in paper posters to deliver a communicable message to the academic and industrial community^1–4^. While the professionals have considerably benefitted from conventional poster presentations, there is an important need to revisit the concepts for posters due to their increasing costs associated with the rapid evolution of research progress and to their inefficacy to incorporate complex datasets such as movies, high dimensional visuals, and two/three dimensional (2D/3D) interactive media.

Here, we propose a “digital poster” concept that can deliver scientific presentations in a paperless format. Digital posters were realized based on a repurposed digital frame platform from the art community. In this paper, the design of digital posters was described from the preparation of digital media to uploading the interactive media to the digital frame via wireless connectivity. The proof-of-concept of digital posters will be demonstrated in the bioimaging data from cellular imaging and movies that are standard media in bioengineering research. The same cellular materials will then be compared to the paper posters and whiteboard based posters. Cost analysis of digital posters in comparison to paper and whiteboard posters will be provided. A futuristic design for a foldable digital poster concept will be presented. The presented digital poster solution could be a transformative interactive tool in other research fields that utilize complex molecular, cellular, organ-level, or structural media.

## From papers to digital posters: Modular and dynamic presentation platform

Digital posters were designed based on digital frames that are emerging platforms in the art field. Artworks stay on display for a long time in the form of pictures in the galleries or individual’s personal locations. An emerging need has been to dynamically change the art-piece at will. A digital art frame was then developed by Meural that is powered by a computer network professional, NETGEAR. This platform enables the upload of the digital copies of art galleries via a wireless network through a computer or a smartphone application. Since it is now widely distributed in the market, the cost is relatively low and affordable by individual users. Therefore, this digital frame platform has provided a perfect fit device for our design for digital posters.

**Fig. 1:**
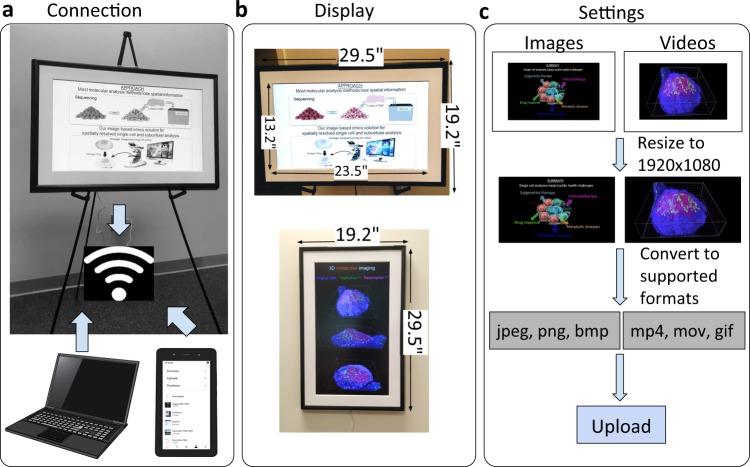
Digital posters: Configuring the device and preparing interactive media. **a** Digital posters are connected to a wireless internet network that is shared with a computer interface and a smartphone application. The display items for posters are received from either a laptop or a handheld smartphone unit. **b** Digital frame can either be vertical or landscape shape and covers a physical dimension of 19.2” L × 29.5” W × 1.6” D with an active screen area of 13.2” L × 23.5” W. This 27” (diagonal) digital frame is then mounted on a poster stand through the two ends of the frame. **c** For optimal visualization of display items in a poster, the image resolution is adjusted to 1920 × 1080 pixels. If the size of the display is small, the screen will not be fully filled. Image formats that are supported for digital posters are .jpeg, .png, and .bmp extensions. Movies and videos are converted to .mp4, .mov, and .gif formats for 2D/3D visuals of cellular imaging datasets. Final display items are then uploaded to the digital frame (Meural) through the application or computer web-interface. Paulista/Shutterstock.com; Alila Medical Media/Shutterstock.com; MicroOne/Shutterstock.com.

After activation of the Meural account for an individual researcher/ meeting organizer, the digital frame was then connected to local wireless connection to synchronize the computer’s digital library and smartphone application’s display galleries (Fig. 1a). The digital frame (Dimensions: 19.2′′ × 29.5′′ × 1.6′′) can be either configured in Portrait or Landscape direction (Fig. 1b). The latter is preferred for digital posters to cover full-page display items. Digital poster materials were then organized using image and video preparation software tools in the format of a poster theme that contains an introduction, approach, methods, results, and summary sections (Fig. 1c). These display items were then uploaded to a specific library that is configured to change at a maximum of every 20 sec. Users can then change the display item earlier than 20 sec using the computer, smartphone, or manual control settings (Supplementary Video 1).

## Setting up the media and digital frames for poster displays

Interactive media can be uploaded to the digital frame using a smartphone (through the Meural app) or through My Meural website on a computer. To send the data to the frame, it must be connected to a Wi-Fi network with a good signal. To connect the frame to the mobile application, initially login to the Meural application and add the Canvas to the device using barcode or the product serial number. Turn on the local network on the Canvas and connect the mobile phone to the local network. Once this connection is established, connect the mobile phone to a Wi-Fi network. The frame will also get connected to this network through the phone. After the frame and the phone are connected to a common Wi-Fi, the mobile phone can be used to control the frame. The app can be used to select the playlist to be displayed, upload media and change properties regarding the frame such as image display time, orientation, among others. Alternatively, the website can also be used to upload media and create, edit and delete playlists.

Before uploading the content to the frame, they must be converted to the supported format and resized to fit the frame (Fig. 1c). The supported formats are.jpg,.jpeg,.png and.bmp for images and.mp4,.mov and.gif for videos. Files of other formats can be converted using online software to the compatible formats. The images and videos must be of the resolution 1920 × 1080 (horizontal) or 1080 × 1920 (vertical) in order to fit the screen. Media content smaller will result in white spaces filling the gap whereas larger images/videos will be cropped out. The best way is to create the original images/videos such that the resolution matches the screen’s resolution. In cases where this is not possible, the content can be resized without loss in image quality using online/offline software.

As previously described, data can be uploaded to the digital frame either using the mobile app or through the website. In both cases, the user has to login in order to be able to connect with the frame. Once logged in, the user can upload images or videos by clicking the “upload” button located on the top of the screen on the right side and selecting the file(s) to be uploaded. After uploading, these files can be combined to create a playlist using the “New playlist” button on the top-right of the screen. The playlist can be named according to the content being displayed and a short description can be added which will be displayed with the playlist’s name. The files are then added to the playlist. Descriptions can be added to individual files also and these will be displayed below the file’s name while viewing on the screen. Once the playlist is finalized, the button that looks like a box with an arrow is clicked and the playlist is sent to the Canvas. The Canvas is reloaded, and the data transfer is verified by displaying the content on the Canvas.

## Digital case study of multiplex cellular bio-images and movies

**Fig. 2:**
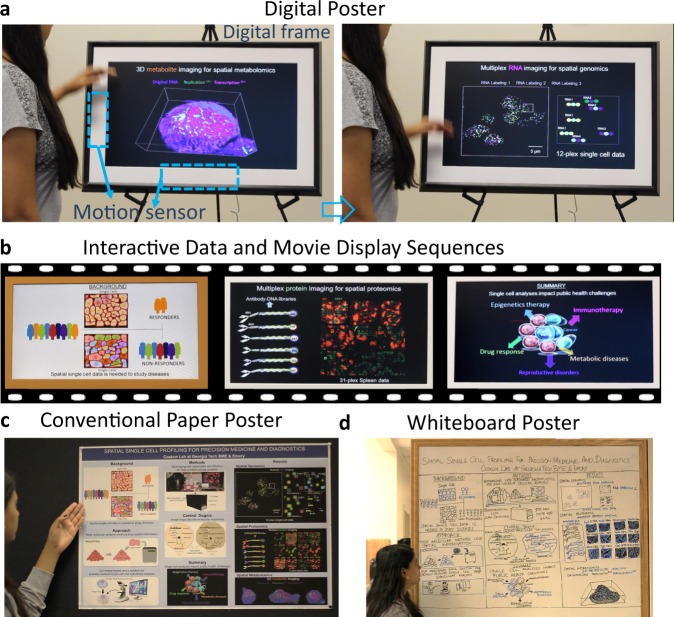
Digital posters for interactive cellular media and comparisons to other poster concepts. **a** A scientific poster in the form of digital frames. Presenters use the motion sensors located at the bottom and left of the screen to change the display item. Digital frames from the artwork field were adapted to poster presentations. A specific album that contains both images and movies from scientific projects was uploaded to the digital posters prior to the presentations. **b** Interactive displays were then displayed in a sequence that is custom formatted in the digital album. Display items were updated by the presenter’s command on the motion sensor, until the maximum display time of previously adjusted 20 sec per display item. One slide at a time was shown in a movie frame out of a total of 9 display items in this specific poster setting. **c** A conventional paper poster was shown for comparison. **d** The same material was also presented on a whiteboard as a classical method. Standard presentation styles, through papers and whiteboards, fall short for dynamic cellular imaging media. MicroOne/Shutterstock.com.

The proof-of-principle of this digital poster was then demonstrated on the bioimaging projects for cellular analysis in health and diseases (Fig. 2a). Nine display items were prepared in a single digital poster album and sequentially displayed with interactive features (Fig. 2b). Presenter waves hand over the motion sensors at the bottom and left locations of the digital frame to change the display item. While different albums can be shuffled in the digital frame settings, a fixed album was preferred under “Spatial single-cell profiling for precision medicine and diagnostics” theme. The first display item was an overview of the other eight display items. The second item was the background information explaining why single-cell profiling is needed to decipher treatment response in distinct patients. The third item was the approach that compared standard molecular profiling that has fallen short on spatial profiling of patient specimens, while the solution presented was to directly visualize molecules in cells without dissociating patient samples in liquid buffers. The fourth item was the method to provide details of the bioimaging platform (Keyence BZ-X810) integrated with microfluidics, followed by a super-computer (Supermicro workstation with 256GB RAM, 2 NVIDIA 2080Ti GPU chips, and 100TB RAID storage) analysis of the resultant cellular imaging datasets. The fifth item was the need to measure molecular conversions across “central dogma” to determine underpinning molecular drivers of disease. These initial five display items were utilized to provide research background one message at a time in large fonts with concise scientific details.

The results section was then introduced with interactive cellular data. The sixth display item was to emphasize “spatial proteomics” methods that can provide molecular distributions of functional proteins at the single-cell level together with cellular positions^5^. One technology to measure multiple proteins in cells is CO-Detection by indEXing (CODEX), wherein DNA barcoded antibodies were used to visualize up to 50-100 molecules as described previously^6, 7^. This technology provides microstructural features of tissues and organs in health and disease. Here, an immune organ known as spleen was shown for 31-markers. T-cell and B-cell enriched regions that are covered by vessels and other cells were shown. To comprehend the complexity of the tissue micro-organization, a 31-plex dataset was played as a movie in this display item with markers shown at the top and colored by the identity of markers in each snapshot of the video. Thus, digital posters have helped visualize this tissue complexity at the cellular level.

Next, the seventh display item was the “spatial genomics” vision that can provide active gene expression of cells in the form of single RNA molecules that provide messages to make sufficient proteins in cellular response^8^. Technologies are available to measure single RNA molecules by fluorescent in situ hybridization (FISH)^9^. However, fluorescent microscopes are limited by technically available colors due to the spectral limitations of devices. Thus, to cover many RNA molecules, molecular barcoding methods have been used to sequentially label the same RNA molecular and decode image video sequences^10^. RNA imaging was performed on a highly sensitive fluorescence microscope (Nikon Ti Eclipse and Olympus IX-81) with single-molecule detection capability using 60× and 100× oil objective lenses^11^. To understand this RNA image barcoding approach, we then played three subsequent labeling schemes for individual RNA molecules that are uniquely colored (cyan, yellow, magenta, green). Here, the co-localization of distinct color changes for the same RNA target was obvious from the digital poster’s display, again providing a powerful platform for informative poster presentations.

Another important advance was in the eighth display item to visualize “spatial metabolomics” data at the subcellular 3D distributions^12^. Cells were enriched or targeted by small molecules that are specific to subcellular features in the cells (replication, transcription, and chromatin) for a short period of time, followed by fixation (chemically trapping the cells)^13, 14^. A mass spectrometry imaging method (NanoSIMS) then resolved subcellular structures and molecular identities pixel by pixels at sub-100-nm resolution. After combining image stacks from depth profiles of cells, 3D subcellular tomograms of metabolically enriched sites were measured. 3D renders of multiple isotopic markers were performed in Imaris (Bitplane) software. To visualize cellular movies, this display item then provided multiple views of a single lymphoblast cell in 3D. Continuous structures and deep structural features were observable in this digital poster, an advancement for poster concept.

The final display item was then the summary of applications that leverage this spatial bioimaging and molecular profiling technologies in various diseases. Interactive details of each disease model can then be extended in later part of an updated digital poster library. Of note, the interactive display items from sixth to eighth would not have reached full depiction and visualizations in the paper poster version of the same spatial imaging datasets (Fig. 2c). Another concept of posters is a whiteboard drawing of the display items (Fig. 2d). While there is more room for customization of display pieces in whiteboard posters, it still fails to provide the depth of interactive cellular images and movies.

## Reducing the cost of poster demands by reusable digital solutions

Research advances at a rapid pace. Once paper posters are printed, results and associated analysis remain unchanged due to the non-editable feature of posters. On average, a single poster costs around $100 when printed at a regular facility. An increasing number of conferences and a growing range of research groups necessitate a linearly expanding scale of posters for an individual researcher (Supplementary Fig. 1a, blue line). On the other hand, the emerging digital posters require a one-time purchase at a fixed cost for the initial stages of poster presentations. One digital frame device should last for a few decades, possibly throughout a researcher’s entire career, as long as the supporting companies and users keep up with the global dynamics of technological advances. In such cases, a driver or software update may be required. For instance, the Meural devices cost around $500 for a single unit and display items for a poster theme can be updated for upcoming meetings. Initial fixed cost is the only required investment and the cost of digital posters remains constant throughout one’s career in academia or industry (Supplementary Fig. 1a, green line). In this scenario, after five different occasions of poster presentations, the fixed cost will be paid off. Thus, digital posters provide a cost-effective option for long-term use with interactive media compatibility.

Another digital poster model is for conference and meeting organizers. For example, organizers invest in ten digital frames for a group poster session. The presenters then would be asked to upload their display items to an online cloud interface, followed by synchronization of each digital poster for the presenter’s materials. Presenters then may be asked to contribute to the initial funds by charging a minimal amount per presentation. After fifty unique presentations, these digital posters also cover the initial costs (Supplementary Fig. 1b). In addition, the availability of digital posters by the organizers alleviates the need for transportation of individuals’ posters from their home locations to meeting venues. This community solution for digital posters reduces the cost of presenters with convenience, adaptability, and interactivity.

## Foldable digital displays for next-generation posters

The next-generation design of digital posters will be in the format of folding displays that are controlled by touchscreen and motion controls (Supplementary Fig. 2). Currently, display manufacturers conceptualize and produce these folding display interfaces at the experimental level. The vision would be to utilize a lightweight, low-cost and interactive folding displays at large size. The presenter then folds the display before and after the poster sessions. Interactive features would also allow presenters to select specific datasets and perform digital analysis directly on the poster surface, providing an active discussion platform between the presenter and the audience. Futuristic digital posters are at their infancy, but the rapid evolution of the display market and reducing costs will make their feasibility possible.

## Digital technologies for bioengineering education

Digital technologies enable innovative instructional platforms. For instance, virtual reality offers training opportunities for students and researchers in engineering and medicine^15–17^. Digital posters, therefore, have great potential for bioengineering education that generates cellular and medical imaging datasets. These emerging platforms stimulated our initiatives for a Bioengineering Media Laboratory (BioE Media Lab), wherein we utilize digital technologies, augmented reality, 3D printing, and cellular imaging art as visual educational themes.

## Supplementary information

Supplementary Information

Supplementary Movie 1

Description of Additional Supplementary Files

## Data Availability

Any display item and related data are available upon request.
