# Supplementary material for: Digital posters for interactive cellular media and bioengineering education
Source: Commun Biol. 2019 Dec 6;2:455. doi: 10.1038/s42003-019-0702-1 (PMC6898652; doi:10.1038/s42003-019-0702-1)
Supplement: Supplementary file 1 — Supplementary Information [file 42003_2019_702_MOESM1_ESM.pdf]

Supplementary Figures

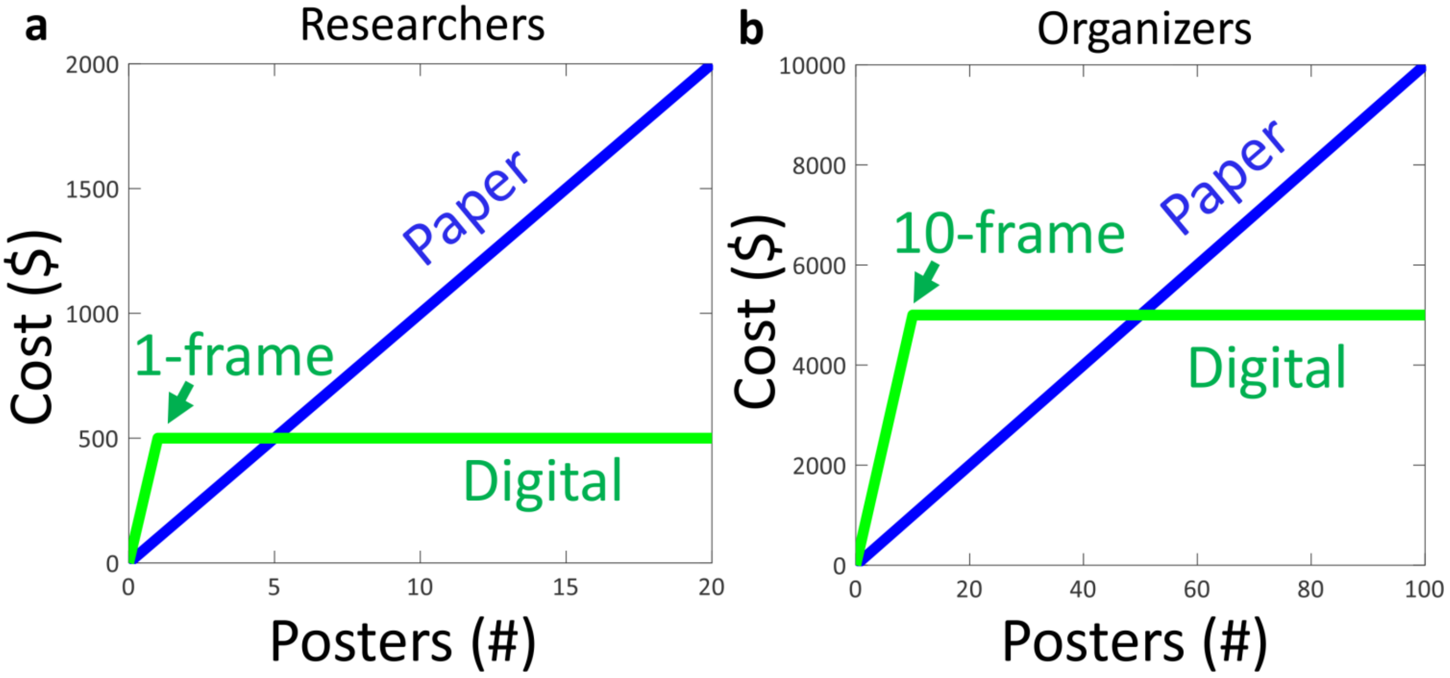

Supplementary Figure 1 | Cost analysis of digital and paper posters. (**a**) Researcher scenario: Poster presentations for individuals increase by the number of conferences and meetings that are lumped into the number of posters (#). An average cost of a single poster is $100. The cost of paper posters linearly increases by poster scale (Blue line). The current cost for a single digital frame is $500, initial investment that is needed for the researcher. After obtaining the device, digital posters remain at a fixed cost (Green line). After five poster presentations, the digital poster pays off its original investment. (**b**) Organizer scenario: Meeting's administrative personnel obtains e.g., ten digital frames for poster presentations. This fixed cost remains constant. Presenters are asked to upload the poster display items to an online cloud. After charging the individual for each usage, the initial cost for these posters will be covered. In longer-term, digital posters provide economical solutions, while the paper posters again scale up linearly in that specific meeting.

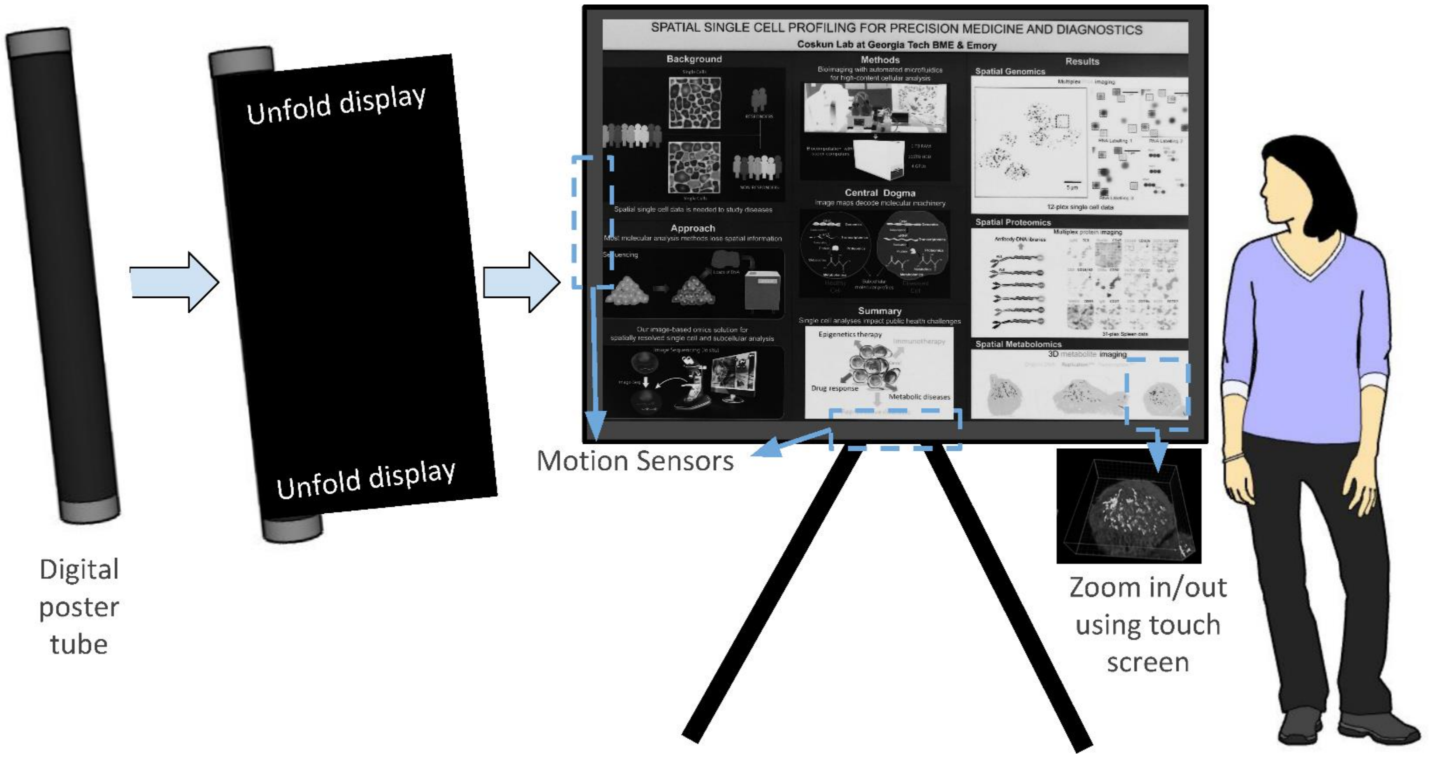

Supplementary Figure 2 | Folding interactive display for next-generation digital posters. Futuristic digital posters will utilize folding digital screens that are wrapped into a tube for portability. Presenter unfolds the folded display and mounts on a poster stand. Motion sensors and active digital touchscreen allows interactions with the display items. Specific regions can be zoomed in and out based on touchscreen commands. After the session, the presenter folds the poster back for transportation.
