# Supplementary material for: Digital posters for interactive cellular media and bioengineering education
Source: Commun Biol. 2019 Dec 6;2:455. doi: 10.1038/s42003-019-0702-1 (PMC6898652; doi:10.1038/s42003-019-0702-1)
Supplement: Supplementary file 3 — Description of Additional Supplementary Files [file 42003_2019_702_MOESM3_ESM.pdf]

Description of Additional Supplementary Files

File Name: **Supplementary Movie 1**

Description: The supplementary movie presents the difference between a conventional paper poster and a digital poster for the digital display items under the theme of "Spatial single-cell profiling for precision medicine and diagnostics". The first part covers a presenter that uses a conventional paper poster to share the research results and data. Conventional posters remain insufficient to visualize dynamic cellular imaging media. The second partshows the same display items as a digital poster using an updatable canvas. Digital posters deliver dynamic media as interactive videos with higher image resolution and quality. The presenter changes the display content using hand gestures over the motion sensors located on the side of the screen. Background Music: « Perception » from Bensound.com. Images: Paulista/Shutterstock.com Alila Medical Media/Shutterstock.com MicroOne/Shutterstock.com.
